# Angiopoietin-2 Levels as Predictors of Outcome in Mechanically Ventilated Patients with Acute Respiratory Distress Syndrome

**DOI:** 10.1155/2017/6758721

**Published:** 2017-08-29

**Authors:** Iraklis Tsangaris, Argirios Tsantes, Eleni Vrigkou, Petros Kopterides, Aimilia Pelekanou, Katerina Zerva, George Antonakos, Dimitrios Konstantonis, Irini Mavrou, Georgios Tsaknis, Evdoxia Kyriazopoulou, Maria Mouktaroudi, Styliani Kokori, Stylianos E. Orfanos, Evangelos J. Giamarellos-Bourboulis, Apostolos Armaganidis

**Affiliations:** ^1^Second Department of Critical Care Medicine, Medical School, National and Kapodistrian University of Athens, Athens, Greece; ^2^Laboratory of Hematology & Blood Bank Unit, Medical School, National and Kapodistrian University of Athens, Athens, Greece; ^3^4th Department of Internal Medicine, Medical School, National and Kapodistrian University of Athens, Athens, Greece; ^4^Department of Biochemistry, Athens University Hospital Attikon, Medical School, National and Kapodistrian University of Athens, Athens, Greece

## Abstract

Pulmonary endothelium dysfunction is a key characteristic of ARDS. The aim of this study was to investigate endothelium-derived markers, such as angiopoietin-2 (Ang-2) and endothelial cell-specific molecule-1 (endocan), at the vascular and alveolar compartments as outcome predictors in ARDS. Fifty-three consecutive ARDS patients were studied. The primary outcome was 28-day mortality. Secondary endpoints were days of unassisted ventilation and days with organ failure other than ARDS, during the 28-day study period. Nonsurvivors presented higher lung injury scores and epithelial lining fluid (ELF) Ang-2 levels compared to survivors, with no significant differences in plasma Ang-2, endocan, and protein C concentrations between the two groups. In logistic regression analysis, ELF Ang-2 levels > 705 pg/ml were the only independent variable for 28-day mortality among the previous four. Plasma endocan values > 13 ng/pg were the only parameter predictive against days of unassisted ventilation during the 28-day study period. Finally, lung injury score > 2.25 and ELF Ang-2 levels > 705 pg/ml were associated with increased number of days with organ failure, other than ARDS. Our findings suggest that Ang-2 levels are increased in the alveolar compartment of ARDS patients, and this may be associated both with increased mortality and organ failure besides lung.

## 1. Introduction

Endothelial barrier damage is a sine qua non of ARDS pathogenesis and a key modulator of ARDS [1]. Therefore, markers of endothelial injury have been the focus of intense research interest, both for their predictive value and as potential treatment targets. Although endothelial-targeted interventions have shown efficacy in several experimental models [2], the distance from bench to bedside seems long. At the same time, prognostication attempts based on a panel of endothelial biomarkers have provided mixed results [3]. The current study addresses this issue, focusing on angiopoietin 2 (Ang-2) and endothelial cell-specific molecule-1 (endocan) in ARDS patients.

Ang-2 is an endothelial growth factor that induces pulmonary polymorphonuclear cell infiltration, enhancing vascular leakage and priming the endothelium to respond to angiogenetic and inflammatory cytokines [4, 5]. Several studies have previously investigated the role of circulating angiopoietins in critically ill patients [6–8]. Specifically, Ang-2 concentrations have been shown to correlate positively with severity of illness scores [7–9] and to hold prognostic potential [7, 9]. Despite this accumulating body of evidence in mixed populations of critically ill patients, the value of Ang-2 as a predictive marker of outcome in the specific population of ARDS has not been fully elucidated [10]. In addition, most studies have measured only plasma Ang-2 levels and focused merely on mortality and not on other patient-centered outcomes [7, 9].

Endocan is a soluble proteoglycan expressed by the vascular endothelium and a key player in the regulation of major processes such as inflammatory disorders [11]. Former studies have shown elevated plasma endocan concentrations in patients with sepsis [12] and have associated them with disease severity and progression [13]. Other studies have identified plasma endocan levels as an independent predictor in septic patients progressing into ARDS [14] and have explored its potential as a biomarker to predict disease severity and mortality in ARDS [15]. Nonetheless, the full value of endocan as a biomarker and the significance of its levels in the endothelial lining fluid (ELF) in ARDS have not yet been studied.

Modulation of the coagulation cascade namely low circulating levels of endogenous anticoagulants and depressed fibrinolysis is thought to contribute to the pathophysiological features of ARDS, and there are increasing evidences suggesting that inflammation and coagulation in ARDS are intimately linked [16, 17]. Alterations in plasma levels of protein C have been associated with mortality and disease severity in ARDS [18], but the significance of these alterations and their association with clinical outcomes have not been elucidated [19]. In addition, since inflammation also modulates endothelial cells to produce plasminogen activator inhibitor-1 (PAI-1), which is the principal regulator of the endogenous fibrinolytic system [20], interest has been raised concerning the importance of the balance between inhibitors and activators of fibrinolysis in ARDS [21, 22].

The aim of this study was to investigate the predictive value of a constellation of endothelial biomarkers, namely, Ang-2, endocan, protein C, and PAI-I for the outcome of a cohort of patients with ARDS. For two of them, Ang-2 and endocan, measurements were also done in ELF that has never been reported in the past. Part of these data has been previously presented in an abstract form at the 24th Annual Congress of the European Society of Intensive Care Medicine [23].

## 2. Methods

### 2.1. Patients

This is a prospective observational study; its protocol was reviewed and approved by our institutional ethics committee. Informed written consent was obtained from the patients' next of kin. The study population consisted of 53 consecutive patients with ARDS treated in the 21-bed mixed medical/surgical ICU of the “Attikon” University Hospital of Athens.

All patients were enrolled within 48 hours of recognition of ARDS. Patients were ventilated with a tidal volume of 6 ml/kg and a combination of PEEP/FiO_2_, according to the NIH protocol [24]. Demographic and clinical data were recorded on study enrollment. Severity indices including acute physiology and chronic health evaluation (APACHE) II, sequential organ failure assessment (SOFA), and lung injury scores were calculated at baseline [25, 26].

### 2.2. Biochemical Studies

We followed the methods of Tsangaris et al., described in detail elsewhere [27]. BAL was performed by fiberoptic bronchoscopy. Six aliquots of 20 ml sterile normal saline were infused through the working channel of the bronchoscope. The first sample of aspirated fluid, reflecting a bronchial sample, underwent microbiological analysis, while the others were collected for biochemical analysis. To avoid free diffusion of urea through the alveolar epithelium, the total recovery time for all aliquots did not exceed 3 minutes. BAL fluid was then filtered through sterile gauze to remove mucus and centrifuged at 3000*g* at 4°C for 15 min. Supernatants were stored at −80°C until assays were performed.

Ang-2 concentration in epithelial lining fluid (Ang-2_ELF) was determined using urea as an endogenous dilution marker, according to the following formula:

(1) $$\begin{array}{c} Ang-2\_ELF=Ang-2\_BAL\times \frac{urea\_SER}{urea\_BAL}, \end{array}$$

where Ang-2_BAL is the measured concentration of Ang-2 in BAL and urea_SER and urea_BAL represent the concentration of urea in serum and BAL, respectively.

Ang-2 levels in plasma and BAL were estimated by an enzyme immunosorbent assay (R&D Minneapolis Mo). The lower limit of detection was 15 pg/ml. An automated chromogenic assay was used for the determination of protein C and an enzyme immunoassay procedure for PAI-1 antigen. Concentrations of endocan in plasma and BAL were measured by an enzyme immunoassay Lunginnov s.a.s (campus de l'Institut Pasteur de Lille, 59000 Lille, France). The lower limit of detection was 0.31 ng/ml.

### 2.3. Blood Samples

Blood samples were drawn just prior to BAL procedure and immediately centrifuged at 3000*g* for 15 min at 4°C (or placed on ice and centrifuged within 1 hour). Plasma was snap frozen in small portions and stored at −80°C until the assays were performed.

### 2.4. Study Endpoints

The primary study endpoint was the identification of the endothelial marker that was associated with at least 80% specificity for unfavorable outcome.

The secondary endpoints were the association of the measured biomarkers with days of unassisted ventilation and with the number of days without organ failure other than ARDS, during the 28-day study period.

### 2.5. Study Power

The study was powered for the primary endpoint. Based on this, and by using the F statistic, to achieve the desired area under the curve of the receiver operator characteristic (ROC) curve with 80% power at the 5% level of significance, it is calculated that a total of 50 patients were needed to be enrolled.

### 2.6. Statistical Methods

Descriptive statistics of the data are presented as means ± standard deviation (SD), medians (Q1, Q3 quartiles), or percentages for normally and nonnormally distributed continuous variables or categorical variables, respectively. Continuous variables were compared between survivors and nonsurvivors by the Mann–Whitney *U* test.

In order to define which of the studied parameters can better discriminate the chances of 28-day mortality, receiver operator characteristic (ROC) curve analysis was done for biomarker. Using the coordinate points of the curve, the point that could discriminate with at least 80% sensitivity was detected and the quantitative variables were transformed into dichotomous variable using this cut-off and compared between survivors and nonsurvivors by the Fisher's exact test. Variables with *p* of comparison below 0.10 entered logistic regression as independent and 28-day mortality as dependent. Odds ratios (OR) and 95% confidence intervals (CI) were calculated. A similar logistic regression analysis was done between these variables with unassisted ventilation after 28 days as the dependent variable. In order to discriminate which of the variables associated with more than 80% sensitivity with 28-day mortality was also associated with organ failures other than ARDS, survival analysis was done. Patients were censored for 28-day mortality, and the number of days without organ failure besides the lung was the endpoint; comparisons were done by the log-rank test.

For hypothesis testing, a *p* value less than 0.05 was considered to be statistically significant. All statistical tests were two-sided. Stata 9.0 software was used for all statistical analyses (Stata Corp., College Station, TX, USA).

## 3. Results

Comparative baseline characteristics of survivors and nonsurvivors are shown in Table 1. Endocan in ELF was below the lower limit of detection. The only differences found were for the lung injury scores, concentrations of ELF Ang-2, endocan, and protein C. More precisely, the lung injury score and the concentrations of Ang-2 in ELF were significantly greater among nonsurvivors. Endocan > 13 ng/ml had a trend for significance to be more common among nonsurvivors. Similarly, there was a trend for significance for protein C to be lower among nonsurvivors.

ROC curve analysis showed that lung injury score more than 2.25, ELF Ang-2 more than 705 pg/ml, circulating endocan more than 13 ng/ml, and circulating protein C lower than 41.5 mg/dl were the best predictors of the 28-day mortality. Logistic regression analysis revealed that the only variable that was associated with the 28-day mortality was ELF Ang-2 greater than 705 pg/ml (Table 2).

Logistic regression analysis for unassisted ventilation during the 28-day study period (Table 3) showed circulating endocan > 13 ng/ml to be the only variable associated with this endpoint. In fact, concentrations > 13 ng/ml were opposing this outcome, that is, high endocan was a driver against unassisted ventilation during the 28-day study period.

**Figure 1:**
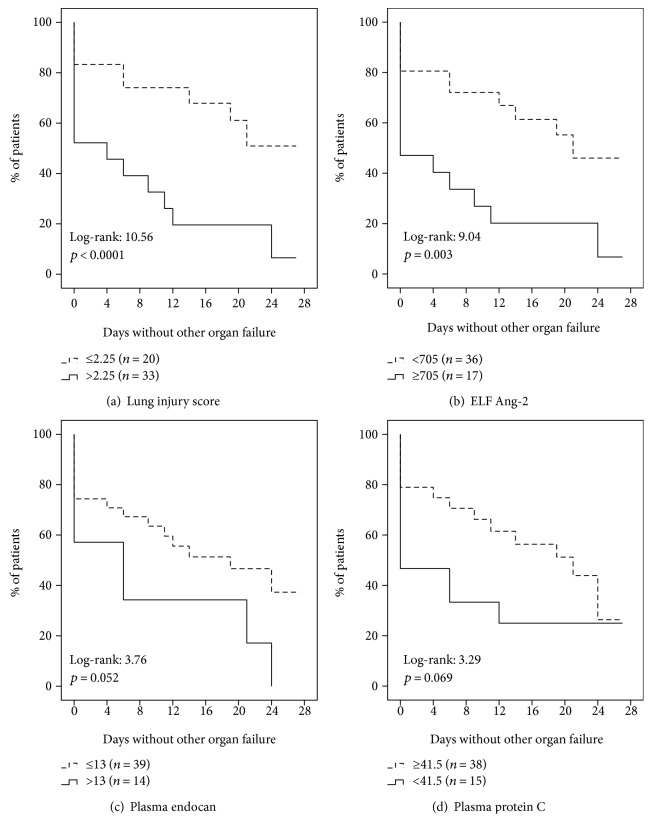
The effect of lung injury score and concentrations of angiopoietin-2 (Ang-2) in epithelial lining fluid (ELF), of circulating endocan, and of circulating protein C on the number of days without organ failure other than ARDS. Survival curves are censored for mortality. The values of the log-rank test and the respective *p* values are shown.

After survival analysis, described in Figure 1, lung injury score more than 2.25 and Ang-2 in ELF more than 705 pg/ml were found to be associated with fewer days without organ failure other than ARDS.

## 4. Discussion

In this prospective clinical study of the prognostic value of endothelial biomarkers, it was shown that ELF Ang-2 was the only independent variable associated with the 28-day mortality. Circulating endocan was the only variable against the number of days of unassisted ventilation during the 28-day study period. Lung injury score and ELF Ang-2 were associated with the number of days with organ failure besides the lung. More precisely, the greater the values these two variables had, the patients presented with more days of organ failure besides ARDS.

Given its role in the disruption of the alveolar-capillary barrier, it is not surprising that Ang-2 has prognostic implications in a mixed population of critically ill patients with ARDS. Patients with lung injury have been shown to have higher Ang-2 levels in pulmonary edema fluid as compared with patients with hydrostatic pulmonary edema [28]. A novel finding of our study is that ELF, and not plasma, Ang-2 levels hold prognostic potential. This should be viewed in the context of similar studies in the field. Gallagher et al. [7] showed in a small study of 18 surgical ICU patients with ARDS that the plasma Ang-2 on the day a patient met criteria for ARDS was higher among the nonsurvivors. Similarly, Ganter et al. [6] found among 208 adult trauma patients that plasma Ang-2 levels are associated with worse clinical outcome. However, none of these studies examined the independent predictive value of plasma Ang-2 in a multivariate model. This was performed by Kumpers et al. [29] in a medical ICU population; in their analysis, only plasma Ang-2 remained significant contrary to lactate, SOFA, and APACHE II scores. Similarly, Ong et al. [9] showed that the plasma Ang-2/Ang-1 ratio was an independent predictor of mortality only in ALI patients with high pulmonary dead-space fraction.

It is not clear why plasma Ang-2, not akin to ELF Ang-2, was not shown to be a predictor of survival in our study. One self-evident explanation is that the previous studies had not tested the value of ELF Ang-2. Another explanation can be that ELF and plasma Ang-2 were not correlated. In this case, they should not be expected to have similar prognostic value. Finally, despite the widespread belief that endothelial cells produce Ang-2, it has been previously shown that airway and alveolar epithelial cells can also express it [28].

A consistent finding for ELF Ang-2 is the association with more days of organ failure other than the lung, making plausible the hypothesis that it is a driver towards systemic inflammation. Endocan is a vasoactive and endothelial-derived molecule that is mainly produced in the event of ARDS. Since previous studies have also associated this biomarker with the progression of septic patients into organ dysfunction [14], it is not surprising that levels greater than 13 ng/ml are predictive for failure to withdrawal from mechanical ventilation.

We did not demonstrate any prognostic value for mortality for protein C and PAI-1. We have previously demonstrated [27] that plasma PAI-1 was negatively associated with ventilator-free days and organ failure in a cohort of ARDS patients partly shared with this study. In a cohort of 549 ARDS patients, Ware et al. [3] showed that protein C and PAI-1 were not among the best performing biomarkers, investigating a panel of plasma biomarkers for predicting mortality.

Our study has a number of limitations that need to be acknowledged. First of all, despite being comparable to similar studies in the field [8, 9], the sample size was small. Therefore, our findings need to be considered as hypothesis generating. Second, we did not measure Ang-1 either in plasma or ELF. The imbalance between Ang-1 and Ang-2 may be more informative than individual markers, [10] but multiple studies have focused on Ang-2 [8, 9] given its preferential expression in the vascular endothelium. Third, we cannot confidently prove if the elevated ELF Ang-2 level was a result of local overexpression or leakage of increased systemic (plasma) Ang-2 from a damaged pulmonary vasculature.

In conclusion, our findings are suggestive of increased Ang-2 levels in the alveolar compartment of ARDS patients that may be associated both with increased mortality and organ failure besides the lung, as well as elevated plasma endocan levels that may hold predictive value against the number of days of unassisted ventilation. Larger studies are warranted in order to explore the significance of these findings and allow proper prognostication of ARDS.

## Figures and Tables

**Table 1:** Baseline demographic and clinical characteristics for survivors and nonsurvivors.

	Survivors (*n* = 25)	Nonsurvivors (*n* = 28)	*p* value
Male gender (*n*, %)	15 (60%)	18 (64.3)	0.783
Age (years, mean ± SD)	63.6 ± 14.1	64.6 ± 16.8	0.822
APACHE II score (mean ± SD)	20.7 ± 5.4	21.5 ± 6.6	0.625
SOFA score (mean ± SD)	7.84 ± 3.70	9.07 ± 3.19	0.200
Lung injury score (mean ± SD)	1.90 ± 0.46	2.48 ± 0.58	<0.0001
Lung injury score > 2.25 (*n*, %)	5 (20.0)	18 (64.3)	0.002
Plasma Ang-2 (pg/ml, median IQR)	7720 (10108.9)	7128 (9232.5)	0.454
Plasma Ang-2 > 18,405 pg/ml (*n*, %)	5 (20.0)	5 (17.9)	1.00
ELF Ang-2 (pg/ml, median IQR)	<4 (<4)	1068.2 (2349.0)	0.001
ELF Ang-2 > 705 pg/ml (*n*, %)	2 (8.0)	15 (53.6)	<0.0001
Plasma endocan (ng/ml, median IQR)	6.15 (9.45)	8.17 (15.45)	0.129
Plasma endocan > 13 ng/ml (*n*, %)	4 (16.0)	10 (35.7)	0.094
ELF endocan (ng/ml, median IQR)	<0.31	<0.31	1.00
Plasma PAI-I (mg/dl), median IQR)	59.0 (71.4)	95.0 (57.2)	0.332
Plasma PAI-I > 140 mg/dl (*n*, %)	4 (16.0)	6 (21.4)	0.732
Plasma protein C (mg/dl, median IQR)	66.2 (42.5)	45.3 (41.65)	0.062
Plasma protein C < 41.5 mg/dl (*n*, %)	4 (16.0)	11 (39.3)	0.074

**Table 2:** Logistic regression analysis of variables associated with the 28-day mortality. Only variables with a *p* value of difference below 0.10 between survivors and nonsurvivors (shown in Table 1) entered the equation.

Variable	OR	95% CIs	*p* value
Lung injury score > 2.25	1.32	0.18–9.29	0.780
ELF Ang-2 > 705 pg/ml	11.18	1.06–117.48	0.044
Plasma endocan > 13 ng/ml	3.36	0.74–15.31	0.117
Plasma protein C < 41.5 mg/dl	3.58	0.73–15.54	0.122

**Table 3:** Logistic regression analysis of variables associated with days of unassisted ventilation during the 28-day study period. Only variables with a *p* value of difference below 0.10 between survivors and nonsurvivors (shown in Table 1) entered the equation.

Variable	OR	95% CIs	*p* value
Lung injury score > 2.25	0.48	0.04–5.55	0.562
ELF Ang-2 > 705 pg/ml	2.69	0.22–33.75	0.441
Plasma endocan > 13 ng/ml	0.09	0.01–0.79	0.030
Plasma protein C < 41.5 mg/dl	0.35	0.07–1.64	0.182
